# Correlations between the clinical, histological and neurophysiological examinations in patients before and after parotid gland tumor surgery: verification of facial nerve transmission

**DOI:** 10.1007/s00405-014-3032-4

**Published:** 2014-04-17

**Authors:** Agnieszka Wiertel-Krawczuk, Juliusz Huber, Magdalena Wojtysiak, Wojciech Golusiński, Piotr Pieńkowski, Paweł Golusiński

**Affiliations:** 1 Department of Pathophysiology of Locomotor Organs, Wiktor Dega Clinical Orthopaedic and Rehabilitation Hospital, University of Medical Sciences, 28 Czerwca 1956r. No 135/147, 61-545 Poznań, Poland; 2 Department of Head and Neck Surgery, Greater Poland Cancer Center, University of Medical Sciences, Poznań, Poland

**Keywords:** Parotid gland tumor surgery, Facial and trigeminal nerve transmission, House–Brackmann scale, Electroneurography and electromyography, Blink reflex

## Abstract

Parotid gland tumor surgery sometimes leads to facial nerve paralysis. Malignant more than benign tumors determine nerve function preoperatively, while postoperative observations based on clinical, histological and neurophysiological studies have not been reported in detail. The aims of this pilot study were evaluation and correlations of histological properties of tumor (its size and location) and clinical and neurophysiological assessment of facial nerve function pre- and post-operatively (1 and 6 months). Comparative studies included 17 patients with benign (*n* = 13) and malignant (*n* = 4) tumors. Clinical assessment was based on House–Brackmann scale (H–B), neurophysiological diagnostics included facial electroneurography [ENG, compound muscle action potential (CMAP)], mimetic muscle electromyography (EMG) and blink-reflex examinations (BR). Mainly grade I of H–B was recorded both pre- (*n* = 13) and post-operatively (*n* = 12) in patients with small (1.5–2.4 cm) benign tumors located in superficial lobes. Patients with medium size (2.5–3.4 cm) malignant tumors in both lobes were scored at grade I (*n* = 2) and III (*n* = 2) pre- and mainly VI (*n* = 4) post-operatively. CMAP amplitudes after stimulation of mandibular marginal branch were reduced at about 25 % in patients with benign tumors after surgery. In the cases of malignant tumors CMAPs were not recorded following stimulation of any branch. A similar trend was found for BR results. H–B and ENG results revealed positive correlations between the type of tumor and surgery with facial nerve function. Neurophysiological studies detected clinically silent facial nerve neuropathy of mandibular marginal branch in postoperative period. Needle EMG, ENG and BR examinations allow for the evaluation of face muscles reinnervation and facial nerve regeneration.

## Introduction

One of the most crucial complications which accompanies parotid gland tumor surgery is the high risk of facial nerve paralysis. Earlier clinical studies [1, 2] and recent research projects of Lim et al. [3] and Barzan et al. [4] indicated the relationship between the type of surgery (surgical technique and its range which is related to tumor type and its location) and different degree of iatrogenic nerve injury. Malignant or benign type of parotid gland tumors is determined by the fine needle aspiration biopsy (FNA), intraoperative evaluation and postoperative histopathological examinations. Imaging like ultrasonography, CT or MRI localize the tumor and allow for its size measurements. Neurophysiological tests precisely analyze the facial nerve function. Comparison of results from all mentioned diagnostics facilitates applying the appropriate treatment. In clinical examination proper function of the facial nerve before surgery cannot exclude its subclinical dysfunction. Electroneurographic studies showed that slowly growing tumors may not evoke the clinical symptoms of the facial nerve paralysis [5]. It was documented by Aimoni et al. [6], that assessment of the facial nerve transmission before surgical removal of tumor is clinically valuable and gives the basis for further postoperative monitoring of its function. The questions arises to what extent the dysfunction in transmission of facial nerve fibers is a consequence of the surgical procedure and if this surgery evokes permanent or temporary changes in nerve function in the long-term observation.

Except for the well-known electroneurographic studies which evaluate extra-cranial transmission in facial nerve fibers [compound muscle action potential (CMAP) analysis], blink-reflex (BR) examination may also be effectively used in neurophysiological diagnostics. It brings the valuable information about afferent transmission in trigeminal nerve, efferent transmission in facial nerve as well as about the ipsi- and contra-lateral polysynaptic connections of brain stem centers and centers at C2–C3 segments of the spinal cord [7] also in patients with facial palsy [8–10]. Needle electromyographical recordings confirmed the face muscles denervation in patients with Bell’s palsy [11, 12], but only early study of Martin and Helsper [13] tried to describe the spontaneous reinnervation of face muscles in the patients after the surgery of parotid gland tumors.

The available literature does not provide descriptions of a combination of the above-mentioned methods in evaluation of such patients. Little is known about correlations between the results from clinical and neurophysiological studies in explanation of subsequent stages of facial nerve pathogenesis in patients with malignant and benign parotid gland tumors preoperatively and with the long-lasting observations.

The aims of this pilot study were: (1) assessment of the histological type of tumor, its size and location in the parotid gland as well as its influence on the facial and trigeminal nerves’ function verified with clinical examination [House–Brackmann (H–B) grading system scale] and with neurophysiological examinations (electroneurography, blink reflex, electromyography) in preoperative period; (2) evaluation of the nerves’ neuronal transmission in postoperative periods (1 and 6 months); (3) assessment of the relationships between results of clinical and neurophysiological examinations.

## Materials and methods

### Patients

Preliminary sample included randomly selected 30 patients (15 women and 15 men aged 16–85 years, 51 ± 18 years on average). Clinical and ultrasonographic examinations and in selected cases CT or MRI examinations confirmed parotid gland tumors and their properties in all patients. Exclusion criteria were previous surgeries of parotid gland (tumor relapse occurred in *n* = 5 patients), head and spine injuries, absence of the patient during the consecutive control studies (*n* = 7), chronic inflammation of parotid gland (*n* = 1). The above criteria excluded 13 patients from the preliminary sample. Final, comparative studies included 17 patients (6 women and 11 men aged 16–71 years, 49 ± 15 years on average) with benign (*n* = 13) and malignant (*n* = 4) parotid gland tumors. The type of tumor was confirmed with postoperative histopathological examinations. Tumor properties are presented in Table 1. Clinical and neurophysiological studies were performed preoperatively once, while postoperative examinations were performed 1 and 6 months after tumor removal.

**Table 1:** Results of histopathological studies and the data on tumor size, their location and type of applied surgery in patients divided in two groups

Tumor types	All patients (*n* = 17)		
I. Benign tumors			
Mixed (pleomorphic adenoma)	9		
Warthin’s (adenolymphoma)	3		
Myoepithelioma	1		
II. Malignant tumors			
Malignant melanoma	1		
Myeloid sarcoma	1		
Carcinoma ex pleomorphic adenoma (pT4apN2bM0)	1		
Salivary duct carcinoma (pT1cN0M0)	1		

Examined group	All patients *n* = 17	Group with benign tumors *n* = 13	Group with malignant tumors *n* = 4
Tumor size (cm)			
1—(small) 1.5–2.4	10	8	2
2—(medium) 2.5–3.4	5	4	1
3—(large) 3.5–4.4	2	1	1
Tumor location in parotid gland			
1—superficial lobe	14	13	1
2—deep lobe	1	0	1
3—both lobes	2	0	2
Type of surgery			
1—extracapsular tumor removal	11	11	0
2—total parotidectomy	4	0	4
3—superficial parotidectomy	2	2	0

Twenty-one healthy volunteers (14 women and 7 men aged 21–51 years, 28 ± 10 years on average) have been examined once in order to estimate the normative values for neurophysiological examinations. In this group H–B score was I. Ethical considerations were in agreement with the Helsinki Declaration. Approval was also received from the Bioethical Committee of University of Medical Sciences (including studies on healthy people). Each patient was informed about the aim of study and gave written consent for examinations and data publication.

### Surgical intervention

All 17 patients were treated by two very experienced surgeons. Facial nerve was dissected in every case of superficial and radical parotidectomy using anterograde or retrograde fashions. In the case of extracapsular approach, facial nerve was dissected when necessary; this was related to the location of tumor within the parotid gland. During surgery, all the patients had their facial nerve activity monitored using NIM Response 2.0 with dual or four-channel system. This monitoring was used for the localization of nerve, nerve’s course mapping and to evaluate its function intraoperatively [14, 15].

**Fig. 1:**
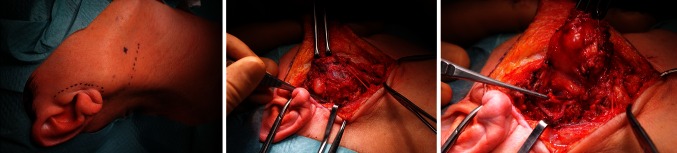
Example of modified Blair’s approach during parotid gland tumor removal

As presented in Table 1 the majority of tumors were small (1.5–2.4 cm) and located in superficial lobe of parotid gland. As far as the type of surgery is concerned extracapsular tumor removal, or superficial parotidectomy surgeries from modified Blair’s approach (Fig. 1) were applied in patients with benign tumor. When the function of nerve is preoperatively normal, it should be preserved during surgery whenever it is possible. Facial nerve scarifying is only required when the tumor invasion into the facial nerve has been confirmed functionally by palsy or histologically. Facial paresis before surgery is an important, negative prognostic factor [16]. In our study, in all four cases of the patients with malignant tumor, it completely encased branches of the facial nerve. In each case the surgeon strived but was not able to totally separate the nerve from the tumor without leaving its macroscopically visible structures. Therefore, radical parotidectomy without facial nerve preservation was applied in all patients with malignant tumor.

### Clinical and neurophysiological examinations

All clinical and neurophysiological examinations were performed by the same investigator. In each patient the facial nerve function on symptomatic and asymptomatic side was evaluated with H–B grading system at every observation period [17–19].

The neurophysiological tests were recorded with Keypoint system (Medtronic A/S, Skovlunde, Denmark). They included facial motor nerve fibers electroneurography (ENG, CMAP), needle electromyography of chosen face muscles (EMG) and BR examinations for evaluation of trigeminal and facial nerves transmission.

**Fig. 2:**
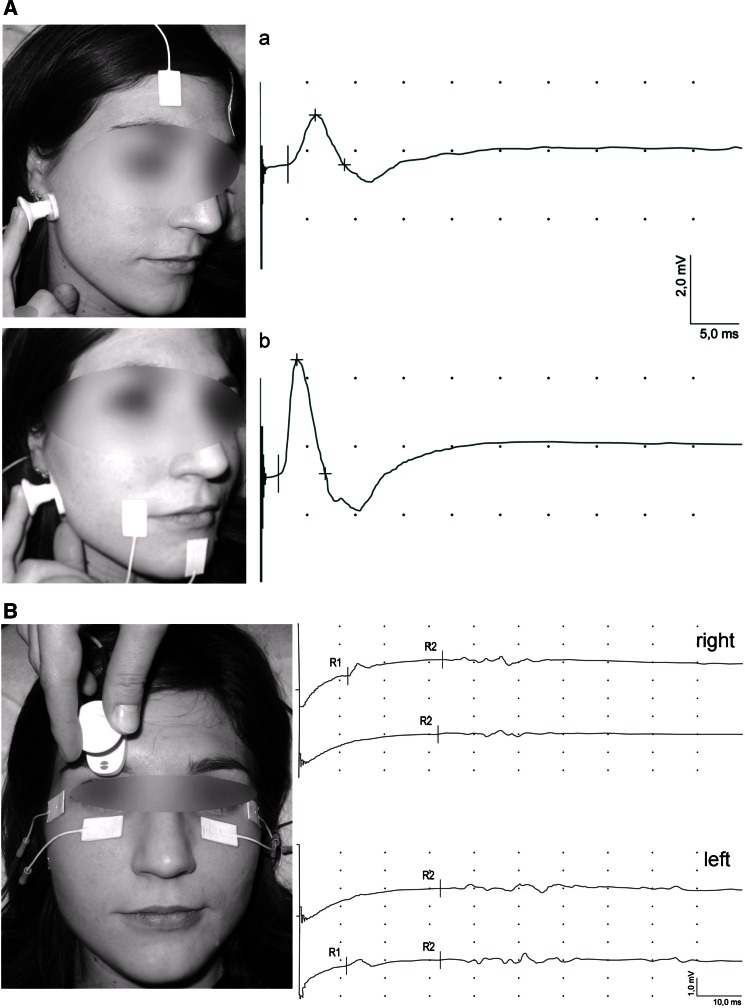
Locations of surface stimulation and recording electrodes during CMAP examinations: **A** from frontal (*a*) and orbicularis oris (*a*) muscles and blink-reflex bilateral recordings; **B** from orbicularis oculi muscles in one of the healthy volunteers

During ENG examinations time base was set on 5 ms/D, sensitivity of recordings on 2 mV/D. 10 Hz upper and 10 kHz lower filters of recorder amplifier were used. Standard (AgCl) surface electrodes were used for recording of compound muscle action potentials (CMAP) evoked from frontal and orbicularis oris muscles. The active electrode was placed on the muscle belly, while reference electrode on the contralateral frontal muscle or on a chin. The electrical stimulation of facial nerve was applied at preauricular area (Fig. 2) [20]. The ground electrode was placed on the neck.

Single rectangular stimuli with a duration of 0.2 ms were delivered via bipolar electrode at 1 Hz frequency, while their intensity ranged from 30 mA to the value evoking the supramaximal CMAP [21]. Parameters of amplitude (measured from negative inflection to baseline in mV), latency (in ms) and standardized latency (in ms/cm) were analyzed. Normative values obtained in a control group for CMAP recordings from the frontal muscle were 1.53 ± 0.42 mV, 3.48 ± 0.55 ms, 0.29 ± 0.04 ms/cm, while from the orbicularis oris muscle were 4.37 ± 1.23 mV, 2.38 ± 0.33 ms, 0.28 ± 0.03 ms/cm. Distance from the stimulating to active recording electrodes was variable due to the different face anthropometric properties of the examined subjects. Calculation of standardized latency per 1 cm included the variability of distance and the proportional variability of latency [22, 23]. The own classification of interpretation the ENG results was applied: 1, normal transmission; 2, mild disturbances in efferent transmission (all parameters of CMAP recorded but different from the norm); 3, severe disturbances in efferent transmission (signs or non-recorded CMAP).

Needle EMG recordings were performed in the patients with the axonal type of facial nerve injury in order to confirm the presence of face muscles denervation at rest as well as to verify the advancement of neurogenic changes during weak muscle’s voluntary contraction. Parameters of motor units action potentials (MUAPs) such as amplitude (mV), duration (ms) and size index (SI, mV/ms) were measured [24]. The assessment of MUAP parameters and the frequency pattern of EMG recording in patients was possible only in the cases when the face muscle voluntary movements were maintained. During recordings calibrations for sensitivity were set at 50 µV/D (at rest) and 500 µV or 1,000 µV/D (during MUAP and frequency analyses) and time base at 20 ms/D and 10 ms or 100 ms/D, respectively. Upper 10 kHz and lower 20 Hz filters of the recorder were used.

During blink-reflex examination the recording electrodes were bilaterally placed over orbicularis oculi muscles (active below lower lid, reference in the area of external eye angle). The ground electrode was placed in the neck (Fig. 2). Bipolar stimulating electrode was applied over the supraorbital foramen. Single rectangular stimuli with duration of 0.5 ms at 1 Hz frequency were used, their intensity was always 20 mA. Supraorbital nerve was stimulated 5–8 times on both sides. Short-latency ipsilateral R1 response consisted with side of stimulation and two long-latency R2 responses were analyzed both ipsi- and contra-laterally [20]. Latencies of recordings in control group were R1 11.06 ± 0.78 ms, ipsilateral R2 30.05 ± 2.54 ms and contralateral R2 30.11 ± 1.79 ms. Similarly as in the interpretation of ENG recordings the own scoring scale was applied.

### Statistical analysis

Shapiro–Wilk’s test was used to establish the normality distribution. The mean values and standard deviations were counted for ordinal scale variables and measurable variables, whereas the frequency of incidence was set for categorical variables. In order to evaluate differences between results obtained in both groups of patients and healthy volunteers, the non-parametric Wilcoxon’s test for dependent variables was used. In the cases of independent variables the non-parametric Mann–Whitney test was used. The level of statistical significance was accepted at *p* ≤ 0.05. Non-parametrical Spearman’s rank correlation coefficient (*r*_s_) was used to demonstrate correlates between ENG results and H–B scores, blink-reflex scores as well as size and type of tumor and type of surgical treatment. For rank correlation *p* ≤ 0.05 was assumed as statistically significant. All statistical analyses were performed with Statistica software version 9.1.

## Results

In the majority of the patients with benign tumors the H–B scores were I at all three observation periods. Only in one patient the H–B scores were VI and IV in the second and third observation periods, respectively (Table 2). Two patients with malignant tumors obtained preoperatively grade III, while postoperative assessment in this group indicated V–VI scores. Facial nerve function on the asymptomatic side in all patients was normal at three periods of observations.

**Table 2:** Data on House–Brackmann scale evaluation in healthy subjects and in patients on the symptomatic side at three periods of observation

Examined group	Control group *n* = 21	All patients *n* = 17			Patients with benign tumor *n* = 13			Patients with malignant tumor *n* = 4		
H–B scale		1st period	2nd period	3rd period	1st period	2nd period	3rd period	1st period	2nd period	3rd period
I	21	15	12	12	13	12	12	2	0	0
II		0	0	0	0	0	0	0	0	0
III		2	0	0	0	0	0	2	0	0
IV		0	0	1	0	0	1	0	0	0
V		0	1	0	0	0	0	0	1	0
VI		0	4	4	0	1	0	0	3	4

H–B scale: *I* normal function, *II* slight dysfunction, *III* moderate dysfunction, *IV* moderately severe dysfunction, *V* severe dysfunction, *VI* total dysfunction

1st, 2nd, 3rd, periods of observation (*1st* before surgery, *2nd* 1 month after surgery, *3rd* 6 months after surgery)

**Fig. 3:**
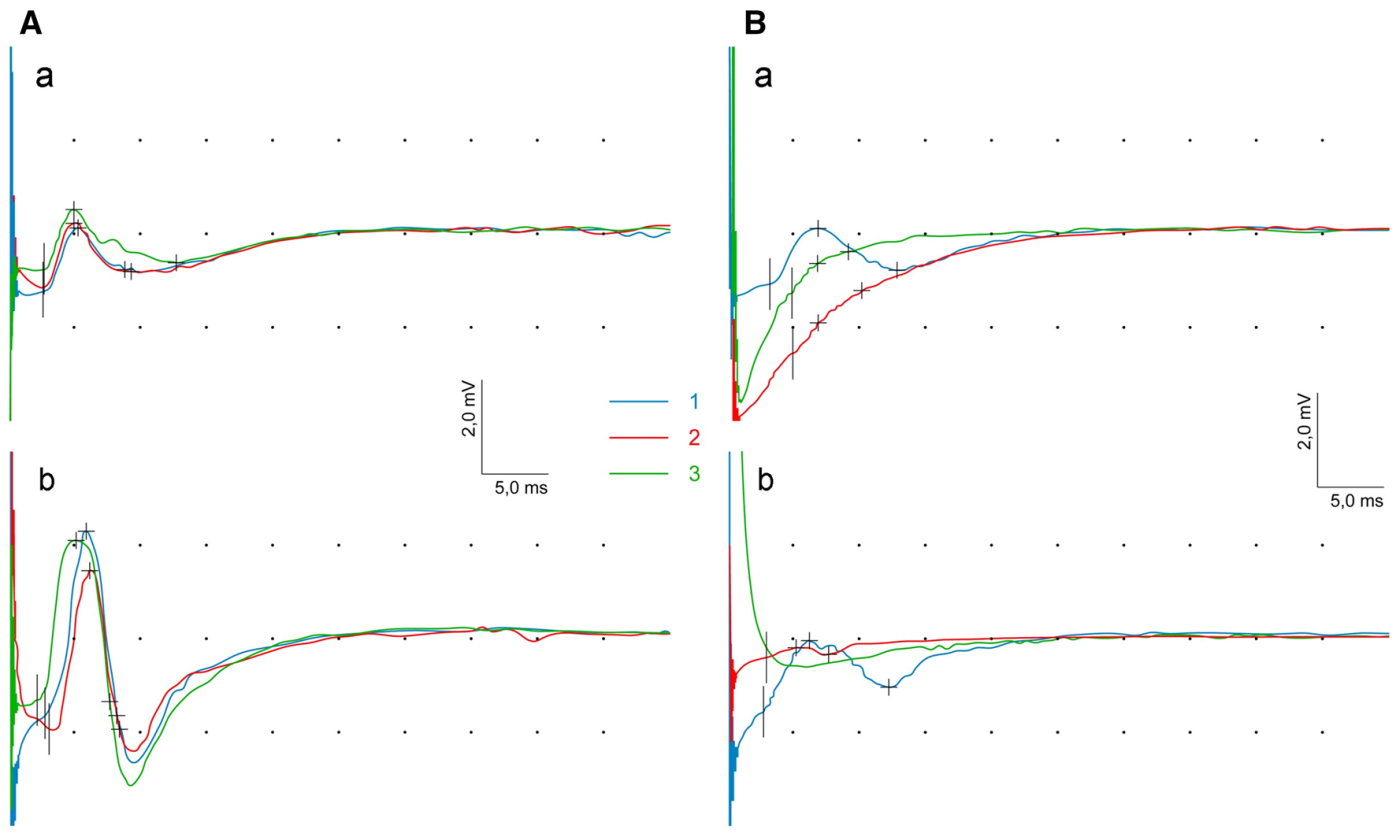
Changes of CMAPs parameters at three periods of observation (1st *blue line*, 2nd *red line*, 3rd *green line*) recorded from frontal (*a*) and orbicularis oris (*b*) muscles in one of patients with benign (**A**) and malignant (**B**) tumors

The CMAP amplitude parameter recorded from orbicularis oris muscle (Fig. 3A, b) significantly decreased at second observation period (upper part of Table 3) in patients with benign tumors. CMAP morphology and latencies when recorded from frontal (Fig. 3A, a) and orbicularis oris muscles (Fig. 3A, b) were comparable at three observation periods as well as in comparison to the asymptomatic side. Significant latency shortening at *p* = 0.009 and increase of standardized latency parameter at *p* = 0.036 in recordings from frontal muscle were present only on asymptomatic side (middle and lower part of Table 3).

**Table 3:** Differences in parameters of recorded CMAP potentials in the group of patients with benign tumors at certain periods of observations

Recorded muscle	Patients with benign tumors 1st vs. 2nd observation (*n* = 13)		Patients with benign tumors 2nd vs. 3rd observation (*n* = 13)	
	Symptomatic side	Asymptomatic side	Symptomatic side	Asymptomatic side
CMAP amplitude (mV)				
Frontal muscle	0.674	0.625	0.529	0.477
Orbicularis oris muscle	0.05*^↑^	0.937	0.168	0.906
CMAP latency (ms)				
Frontal muscle	0.695	0.009*^↓^	0.722	0.266
Orbicularis oris muscle	0.224	0.382	0.248	0.953
CMAP standardized latency (ms/cm)				
Frontal muscle	0.760	0.314	0.154	0.036*^↑^
Orbicularis oris muscle	0.721	0.894	0.147	0.086

Significant differences found at *p* ≤ 0.05 are marked with asterisks (*). Arrows indicate increase↑ or decrease↓ of analyzed parameters

**Fig. 4:**
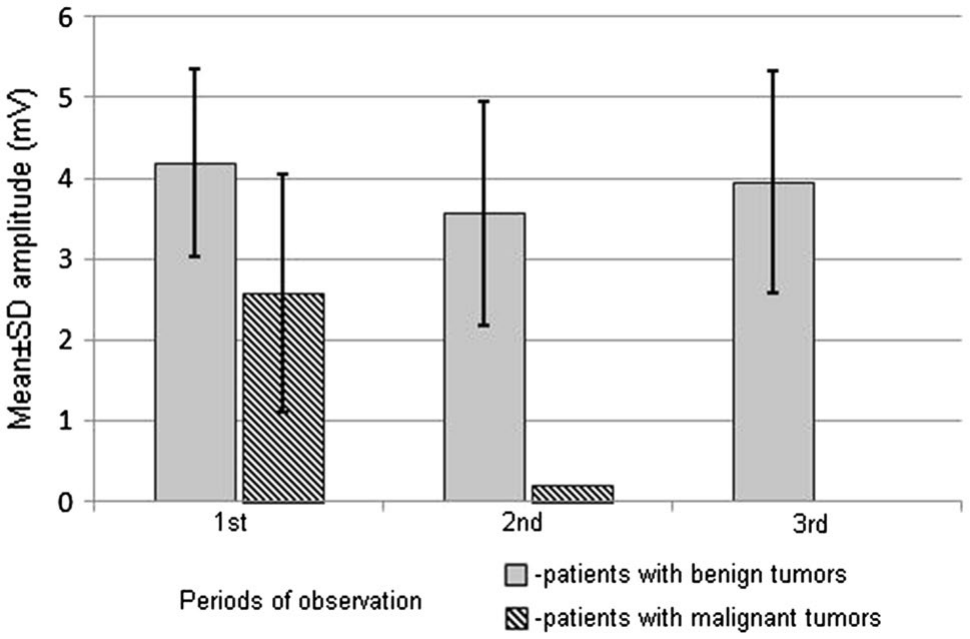
Changes of mean values of CMAP amplitudes recorded from orbicularis oris muscles in patients with benign and malignant tumors at three periods of observations

Patients with malignant tumors at second and third periods of observation showed decrease of CMAP amplitude or lack of CMAP on symptomatic side (Fig. 3Ba, b). Particularly, potentials from orbicularis oris muscles were not recorded (Fig. 4).

Mean values of latencies in R1 and R2 responses of BR at three observation periods of patients with benign tumors did not differ significantly from control values and contralateral BR parameters.

**Fig. 5:**
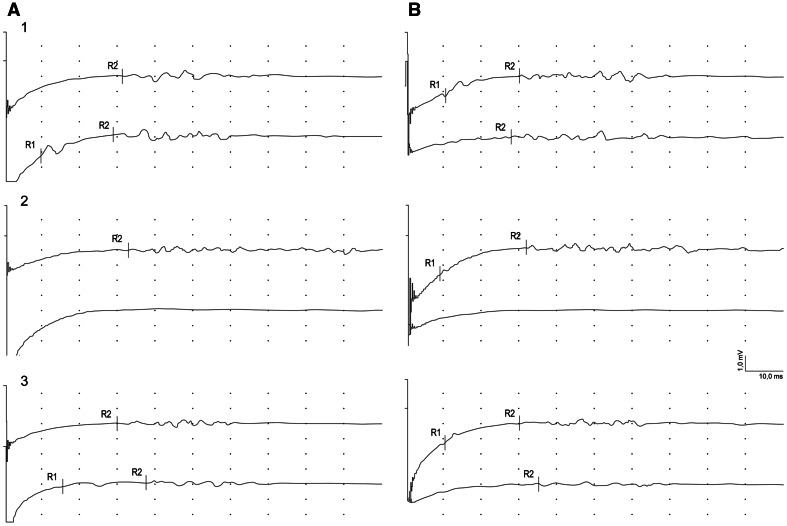
Examples of blink-reflex recordings from symptomatic (**A**) and asymptomatic (**B**) side in one of patients with benign tumor at three (1–3) periods of observation

The example of blink-reflex recordings in one of the patients with benign tumor is shown in Fig. 5. Recordings performed 1 month after the surgery showed no R1 and R2 ipsilateral responses following the stimulation on symptomatic side (A) and no R2 contralateral response after stimulation on asymptomatic side (B). Such a result reflects the iatrogenic injury of the facial nerve. The appearance of the above-mentioned responses following regeneration of facial nerve can be observed at the third period of observation (lowermost recordings).

In patients with malignant tumors, there was a tendency for prolonged latencies of R1 and R2 responses recorded preoperatively on symptomatic side suggesting peripheral demyelinating changes not observed in patients with benign tumors. No BR responses detected postoperatively indicated total facial nerve injury.

**Fig. 6:**
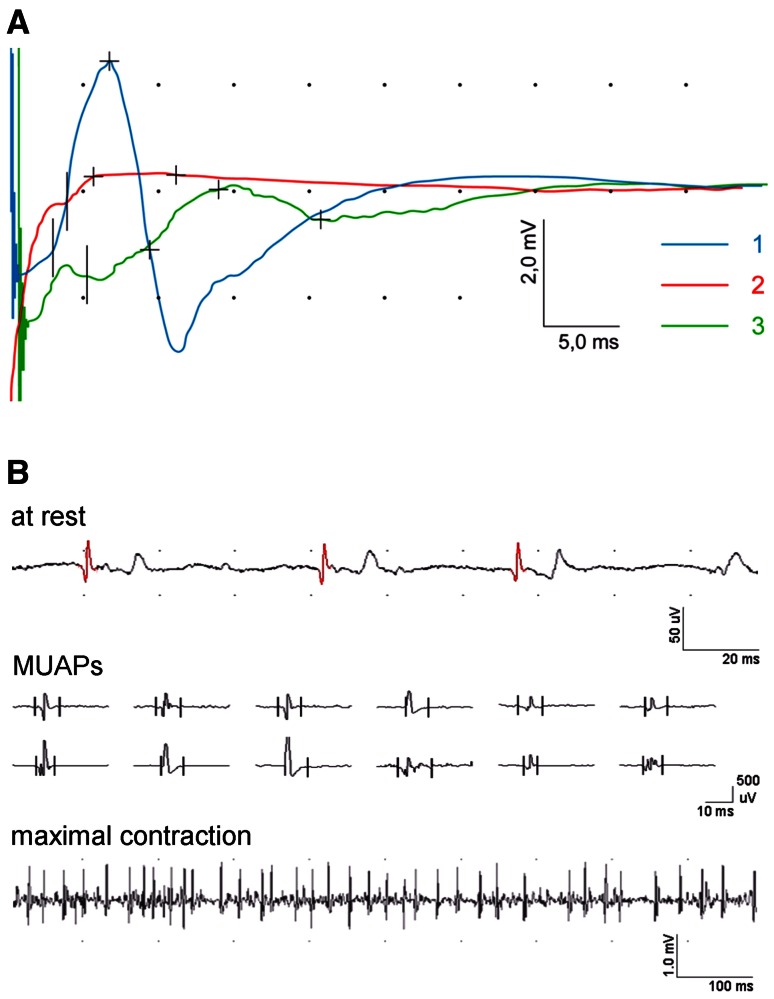
Examples of CMAP recordings: **A** at three periods of observation (1st *blue line*, 2nd *red line*, 3rd *green line*) and **B** needle EMG recordings from orbicularis oris muscle in one of patients with benign tumor proving the regeneration and reinnervation processes

The progress of regeneration in facial nerve fibers is presented in Fig. 6 for one of the patients with benign tumor. The low-amplitude CMAP (A) at the second period of observation and both fibrillations and positive sharp waves in EMG recordings from orbicularis oris muscle were recorded. The third observation period revealed the occurrence of CMAP with larger amplitude and prolonged latency, which indicated regeneration of motor fibers. This phenomenon led to the muscle’s reinnervation which was manifested by disappearing of positive sharp waves with still observed fibrillations, increased parameter of MUAPs duration and low-frequency EMG pattern (B).

Pre- and post-operative EMG recordings at rest showed denervation potentials mostly in the patients with malignant tumors (Table 4). No voluntary activity of motor units was recorded from frontal and orbicularis oris muscles in these patients. Needle EMG examinations were restricted to the muscle analysis at rest because of lack of the face muscles voluntary activity. In general, ENG examinations did not show axonotmesis in facial nerve in the patients with benign tumors, and for those reasons needle EMG examinations were not performed.

**Table 4:** Data on denervation potentials in face muscles at rest during needle EMG recordings at subsequent periods of observations in 17 patients

Observation periods	1st period		2nd period		3rd period	
Patients groups	Frontal muscle	Orbicularis oris muscle	Frontal muscle	Orbicularis oris muscle	Frontal muscle	Orbicularis oris muscle
Benign tumors *n* = 13	− 0/13	− 0/13	+ 1/13	+ 1/13	+ 1/13	+ 1/13
Malignant tumors *n* = 4	+ 2/4	+ 2/4	+ 4/4	+ 4/4	+ 4/4	+ 4/4

For denervation “activity” “+”present, “−” absent

Correlations (at *p* ≤ 0.05) were found between the type of tumor and the results of ENG examinations and results of H–B scoring and ENG outcomes in preoperative period (Table 5). In both cases *r*_s_ coefficient indicated high positive correlations. The average relationship at *r*_s_ = 0.481 was found between the results of ENG studies and the type of surgeries in 1 month time after the procedure. High positive correlations between ENG and H–B or BR outcomes were found in the second and third postoperative periods of observation.

**Table 5:** Spearman’s rank correlations (*r*_s_) of test results and tumor characteristics as well as types of surgeries obtained on the affected side in all patients at three stages of observation

All patients *n* = 17						
Periods of observation and parameter	1st period		2nd period		3rd period	
	*r*_s_	*p*	*r*_s_	*p*	*r*_s_	*p*
ENG (1–3 score)						
Tumor size (1–3 score)	0.347	0.158	Not compared	Not compared	Not compared	Not compared
ENG (1–3 score)						
Tumor type (I–II)	*0.862*	**<0.001**	Not compared	Not compared	Not compared	Not compared
ENG (1–3 score)						
Surgery type (1–3)	Not compared	Not compared	0.481	**0.043**	Not compared	Not compared
H–B scale (I–VI)						
ENG (1–3 score)	*0.837*	**<0.001**	*1.000*	**0.000**	*1.000*	**0.000**
BR score (1–3)						
ENG (1–3 score)	0.425	0.079	*0.738*	**<0.001**	*0.792*	**<0.001**

For ENG and BR scores: 1, normal transmission; 2, mild disturbances in transmission (all parameters recorded but different from normatives) 3-severe disturbances in transmission (signs or potentials not recorded). Abbreviations are the same as in Tables 1 and 2. Italicized values indicate significant results with different power

*p *< 0.05 has been assumed for rank correlation as statistically significant (values in bold)

## Discussion

Neurophysiological assessment of the facial nerve function constitutes a particularly important evaluation in the cases of parotid gland tumor because of the anatomical integrity of the nerve and the gland. Such a study allows for ascertaining the motor fibers transmission following the injury’s advancement or remission [25]. Objectivity of this test depends on the methodological regime and its performance by the same person, especially in prospective studies [26].

Similar to the studies of Yang et al. [27], we confirmed with H–B score the rare phenomenon of facial nerve injury after benign parotid gland tumor removal (Table 2). We observed that the postoperative facial nerve injury depended mainly on the tumor grade of malignancy which determines the surgery’s radicality. Such a phenomenon was also presented by Ruaux et al. [28]. The data shown in Table 5 indicate the positive correlation between changes in facial motor fibers transmission found in ENG and the type of tumors and surgeries.

Danielides et al. [29] as well as Beck and Hall [30] found the relationship between changes in facial nerve function detected in ENG studies and clinical H–B scoring in 97 % of the patients with Bell’s palsy. We confirmed this high positive correlation in patients with parotid gland tumors at three periods of observation like Bendet et al. [5] and Aimoni et al. [6]. However, it should be taken into consideration that clinical H–B scoring assesses all facial nerve branches generally without the possibility of selective injury evaluation.

The ENG examination allows for the study of separate branches of the facial nerve, which leads to the detection of the individual, functional changes. This is illustrated by the data presented in Table 3 showing the significant decrease of CMAP amplitude during postoperative recordings from orbicularis oris muscle in patients with benign tumors following stimulation of the marginal mandibular branch. The nerve branch is described in literature as the most frequently injured during parotid gland tumor removal [31, 32]. In our study the amplitude decrease was reached 25 %, which reflects the same number of axons loss. These values can suggest neuropraxia or mild axonotmesis which was not manifested, however, in the clinical study (score I in H–B scale). This may also indicate the higher sensitivity of neurophysiological assessment than H–B scoring in evaluation of subclinical changes in facial nerve function. This result confirms the conclusion of Esslen [33] that the apparent facial nerve palsy may appear at 50 % of axon loss. Aimoni et al. [6] explained CMAP amplitude deficits by ischemic changes or edema within the nerve. The comparison of this parameter between the second and third period of observation did not show changes, which suggests the lack of pathology regression or progression and indicate rather loss of axons than edema. The radicality of surgery seems to be the best explanation of no CMAP recording in the patients with malignant tumor (Fig. 4), as results of severe damage or complete nerve degeneration and the loss of a chance for a spontaneous regeneration.

Changes in latencies of CMAP evoked on the asymptomatic side (Table 3) seem to be related to the variable distance between stimulation and recording points because of the anthropometric differences among patients. This is proved by the stable standardized latency compared between the first and second observation periods. Statistically significant differences in values of the standardized latency between the second and third period of observation reached only 0.02 ms/cm, which militates for the minimal measurement influence on the final result.

Denervation activity or neurogenic changes in face muscles recorded by EMG confirms axonal changes in facial nerve regardless of the injury reason [11, 25]. In our pilot study (Table 4), EMG was performed mainly in patients with malignant tumors and it was limited to evaluation of the muscle at rest with recordings of denervation potentials. Lack of face muscles voluntary activity did not allow recording of MUAPS because of iatrogenic facial nerve injury caused by the radical parotid tumor surgery. In the cases of patients with benign tumors the chance for saving the nerve’s anatomical continuity is greater, which allows for the spontaneous nerve regeneration and reinnervation processes in face muscles (Fig. 6). Similar to the study of Martin and Helsper [13], we confirmed that the recovery of face voluntary activity is a good prognostic symptom of facial nerve regeneration.

Blink-reflex examinations, despite their diagnostic value, are not commonly used in pre- and post-operative diagnostics of the patients with parotid gland tumors. Besides the fact that BR undoubtedly provides valuable information on the facial nerve function, the assessment of trigeminal nerve in patients with malignant tumors during this study did not have particular diagnostic meaning. The pathology in the efferent branch of reflex arc (facial nerve) masks the function of afferent branch (trigeminal nerve). Proper transmission of nerve impulses in afferent branch can be only recognized from recording of contralateral R2 response with correct parameters. Data presented in Table 5 indicate high correlation between BR and ENG results in evaluation of facial nerve function in the second and third periods of observation. Such an important connection was not observed in preoperative study, which can be explained by the greater sensitivity of BR than ENG examination, what was also underlined by Manca et al. [9] and Mikula et al. [10].

Face, from a psychological point of view, is considered to be the most important part of the body. Permanent facial nerve paralysis is the most devastating complication which is hard to accept for the patient and highly challenging for the surgeon. Facial palsy is associated with impairment in expressing emotions, causes difficulties with speech, intake of food and fluids. As a consequence, it may lead to reduced self-esteem, increase of anxiety, depression and social isolation, and generally lowering the quality of life [34].

## Conclusions

The patients with parotid gland tumors should undergo multidisciplinary diagnostics both in pre- and post-operative periods. Neurophysiological studies allow for selective functional assessment of each facial nerve branch which is crucial for parotid gland surgery. They allow undertaking decision on the surgical nerve reconstruction at appropriate time [35].

As it was presented in this pilot study, the symptoms of silent facial nerve neuropathy are visible in the results of neurophysiological examinations. The range of the facial nerve injury is determined by the tumor type and the surgical procedure. EMG, ENG and BR examinations allow for the evaluation of face muscles reinnervation and facial nerve regeneration. Taking into account difficulties in selection of the homogenous group of patients with malignant tumors because of the rare epidemiology and the applied exclusion criteria, further study should be undertaken to confirm results presented in this study.
